# STK32C as a Therapeutic Target in Colorectal Cancer via HSP90-PI3K/AKT/mTOR Signaling

**DOI:** 10.7150/ijbs.121647

**Published:** 2025-09-29

**Authors:** Chi-Hoon Ahn, Ji Eon Park, Deok Yong Sim, Su-Yeon Park, Hyun Ju Cha, Bum-Sang Shim, Bonglee Kim, Sung-Hoon Kim

**Affiliations:** 1 Cancer Molecular Targeted Herbal Research Laboratory, College of Korean Medicine, Kyung Hee University, 1 Hoegi-dong, Dongdaemun-gu, Seoul 02447, South Korea.; 2 South Baylo University, Suite 500, 4055 Wilshire Blvd, Los Angeles, CA90010, USA.

**Keywords:** STK32C, Colorectal cancer, Apoptosis, HSP90, PI3K/AKT/mTOR.

## Abstract

Emerging evidence implicates serine/threonine kinase 32C (STK32C) overexpressed in bladder cancer and brain tissues acts as a molecular target for doxorubicin resistance, yet its role in colorectal cancer (CRC) remains unclear. Thus. this study investigates the oncogenic mechanism of STK32C in CRC and its interplay with HSP90 and the PI3K/AKT/mTOR signaling axis. STK32C was markedly upregulated in CRC cell lines (HCT116, HT29, SW480, SW620) compared to normal fibroblasts (CCD-18Co) with poor prognosis. STK32C depletion suppressed proliferation, migration, and invasion, while promoting apoptosis—as evidenced by increased Bax, Annexin V, TUNEL-positive, and sub-G1 populations, alongside reduced Bcl-2, pro-Caspase-3, and pro-PARP. Mechanistically, STK32C directly bound the N-terminal domain of HSP90, as shown by immunoprecipitation, immunofluorescence, and GST pulldown assays. Consistently, STK32C depletion or HSP90 N-terminal inhibitor Ganetespib reduced STK32C and p-AKT1, while the HSP90 C-terminal inhibitor, epigallocatechin gallate (EGCG) or AKT inhibitor LY294002 did not affect STK32C, implying that STK32C acts as an upstream of AKT. Furthermore, STK32C depletion enhanced 5-fluorouracil (5-FU) efficacy, with synergistic effects confirmed by CompuSyn and SynergyFinder analysis. *In vivo*, STK32C depletion reduced the growth of HCT116 cells in BALB/c mice with decreased expression of STK32C, HSP90, PCNA, and AKT and activated caspase 3. Overall, these findings suggest STK32C as a novel oncogenic driver in CRC that modulates HSP90 and PI3K/AKT/mTOR signaling and highlights its potential as a therapeutic target alone or in combination with 5-FU.

## Introduction

Colorectal cancer (CRC) is the third most commonly diagnosed cancer and ranks second in cancer-related deaths worldwide^1^. CRC progression is initiated by primary tumor formation caused by uncontrolled proliferation of cancer cells via anti-apoptosis signaling^2^, ^3^, followed by migration or invasion into the surrounding tissue, dissemination and extravasation mainly into liver, lungs, brain, and peritoneum^4^, ^5^.

Although standard chemotherapies—particularly 5-fluorouracil (5-FU)—have improved patient outcomes, drug resistance remains a critical clinical challenge^6^, ^7^. Thus, combination therapy has therefore attracted significant attention as a strategy to reduce chemoresistance and enhance antitumor effect of anticancer agents including 5-FU to induce genotoxic stress in tumor cells^8^, ^9^. Supporting this notion, Kumar *et al.*^10^ proposed a new paradigm in which paradigm in which siRNA-based gene silencing could be combined with conventional chemotherapy to improve therapeutic outcomes.

Among oncogenic networks implicated in CRC, the PI3K/AKT/mTOR (PAM) axis is one of the most frequently dysregulated pathways in several cancers, contributing to tumor growth, metastasis, and poor response to treatment^11^. Thus, dysfunction of this signaling pathway is reported to induce treatment resistance and tumor progression via PTEN loss^12^, PI3K hyperactivation^13^, or AKT overexpression^14^ in several cancers.

In parallel, the molecular chaperone heat shock protein 90 (HSP90) consisting of three main conserved domains known as the N-terminal domain, middle domain, and the C-terminal domain^15^ plays a crucial role in stabilizing numerous oncogenic client proteins, including AKT^16^, thereby reinforcing survival signaling in CRC cells^11^, ^17^. Thus, inhibition of HSP90 destabilizes client proteins such as AKT and 3-phosphoinositide-dependent protein kinase-1 (PDK1)^18^, disrupting PAM signaling and sensitizing tumor cells to apoptosis. Because HSP90 supports tumor proliferation, invasion, and survival, HSP90 inhibitors are considered attractive therapeutic options in CRC^19^, ^20^. Indeed, Basso *et al.*^16^ demonstrated that functional HSP90 is required for Akt stability, as intracellular Akt forms a complex with HSP90 and CDC37, regulated by PI3K activity.

In this oncogenic context, serine/threonine kinase 32C (STK32C), also known as YANK3, has recently emerged as a potential regulator of drug resistance and cancer cell metabolism^21^. STK32C belongs to the AGC kinase family, which includes well-established oncogenic kinases such as 3-phosphoinositide-dependent protein kinase 1(PDK1)^18^, protein kinase B (PKB)/Akt, large tumor suppressor (LATS) and aurora kinases^22^. While STK32C is known to be overexpressed in brain tissue^23^ and bladder cancer^24^, the underlying oncogenic role of STK32C remains undefined in CRCs to date. Importantly, recent findings indicate that STK32C contributes to doxorubicin resistance in triple-negative breast cancer by modulating glycolysis^21^, suggesting its broader role in tumor progression and drug response. Our preliminary analysis demonstrates that STK32C is markedly overexpressed in CRC cell lines compared to normal fibroblasts, highlighting its potential as a novel therapeutic target. Moreover, another STK family member, STK33, has been reported to promote RAS-dependent tumor survival and chemotherapy resistance^25^, ^26^, indicating the importance of this kinase family in modulating treatment sensitivity, including 5-FU.

Based on these evidences, the present study aims to elucidate the oncogenic role of STK32C in CRC via its regulatory interaction with HSP90 and PAM signaling axis. We also investigate whether inhibition of STK32C can potentiate the antitumor effects of 5-FU, thereby positioning STK32C as a molecular target to enhance chemotherapy in colorectal cancer.

## Material and Methods

### Cell culture

Human colorectal cancer cell lines HCT116, SW480, SW620, and HT29 were purchased from the American Type Culture Collection (ATCC). All cancer cells were cultured in RPMI 1640 medium (Welgene, Gyeongsan, Korea) supplemented with 10% fetal bovine serum (FBS) and 1% antibiotic-antimycotic solution containing penicillin, streptomycin, and amphotericin B. Normal colon fibroblast CCD-18Co cells were cultured in Dulbecco's Modified Eagle's Medium (DMEM) supplemented with 10% FBS and 1% antibiotic-antimycotic solution. All cells were maintained at 37 °C in a humidified atmosphere containing 5% CO₂ and confirmed to be free of mycoplasma contamination.

### RNA interference

Cells seeded overnight were transfected with STK32C siRNA, overexpression plasmids, or negative control plasmids (Bioneer, Daejeon, Korea) using INTERFERin® reagent (Polyplus, Bas-Rhin, France) in 200 μL volume, following the manufacturer's protocol.

### TCGA data analysis and Kaplan-Meier survival

STK32C mRNA expression in colorectal cancer patient samples was analyzed using The Cancer Genome Atlas (TCGA) dataset with R Studio software. Disease-free survival (DFS) and overall survival (OS) rates were calculated using the Kaplan-Meier method.

### Tissue microarray and immunohistochemistry (IHC)

Human colon tumor tissue microarrays containing 7 malignant and 2 normal samples were obtained from Biomax (MD, USA). Tissue sections (5 μm) were fixed with 4% paraformaldehyde, dehydrated, embedded in paraffin, and stained with anti-STK32C antibody (1:300) at 4 °C. Detection was performed using DAB substrate, and slides were imaged by Leica SCN400 (Leica, Hesse, Germany). IHC scoring was quantified by ImageJ software, integrating staining intensity and percentage of positive cells.

### Cytotoxicity assay

HCT116 and SW620 cells transfected with control or STK32C siRNA were seeded in 96-well plates (1×10⁴ cells/well). After incubation for 24, 48, or 72 hours, cells were treated with 20 μL MTT solution (1 mg/mL) for 2 h at 37 °C. Formazan crystals were dissolved in 100 μL DMSO, and absorbance was measured at 570 nm using a microplate spectrophotometer (Bio-Rad, CA, USA).

### Colony formation assay

Transfected HCT116 and SW620 cells (1×10³ cells/well) were seeded in 6-well plates and cultured for 2 weeks, with medium changed every 3 days. Colonies were stained with Diff-Quik solution 2 (Sysmex, Kobe, Japan) and counted under a stereomicroscope.

### Wound healing assay

HCT116 cells transfected with control or STK32C siRNA were scratched with a plastic pipette tip to create a wound. After 72 h incubation, cell migration into the wound area was assessed microscopically.

### Migration and invasion assays

Transwell inserts with 8 μm pores (Costar, MD, USA) were used. For invasion assays, inserts were coated with 100 μL Matrigel (Corning, NY, USA). Cells (1×10⁵) were seeded in serum-free medium in the upper chamber; medium with 10% FBS was added to the lower chamber. After 24 h at 37 °C, migrated or invaded cells were fixed and stained with Diff-Quik. Quantification was performed using inverted microscopy and RTCA Software Pro.

### Cell cycle analysis

Cells transfected with siRNA were fixed overnight in 70% ethanol at -20 °C. After RNase A treatment (1 mg/mL, 37 °C, 50 min), cells were stained with propidium iodide (50 μg/mL) at room temperature and analyzed by flow cytometry (FACSCalibur, BD Biosciences).

### cDNA microarray and data analysis

Total RNA was extracted from transfected HCT116 cells using TRIzol reagent and quality assessed with RNA 6000 Nano Chip (Agilent Technologies). Libraries were prepared using QuantaSeq 3' mRNA-Seq Library Prep kit (Lexogen) and sequenced. Differential gene expression and pathway analysis were conducted using DAVID and Medline databases. Experiments were performed in triplicate and repeated with the help of e-Biogen Corporation (Seoul, Korea).

### TUNEL assay

Apoptotic cell death was detected with DeadEnd™ TUNEL system (Roche) following manufacturer's instructions. Cells were fixed, permeabilized, labeled, and visualized by FLUOVIEW FV10i confocal microscopy (Olympus).

### Western blotting

Cell lysates were prepared using RIPA buffer with protease and phosphatase inhibitors. Protein concentrations were measured by Bio-Rad DC assay. Proteins were separated by SDS-PAGE, transferred to nitrocellulose membranes, blocked with 5% skim milk, and incubated with primary antibodies (listed in main text) followed by HRP-conjugated secondary antibodies. Detection used ECL reagent and images were acquired with Amersham Imager 600.

### Real-time quantitative PCR (RT-qPCR)

Total RNA was extracted with QIAzol (Qiagen). cDNA synthesis was performed using oligo dT primers and M-MLV Reverse Transcriptase. qPCR was run on LightCycler (Roche) with primers specified in the main text. GAPDH was used as endogenous control.

### Co-immunoprecipitation (Co-IP)

Whole-cell lysates (500 μg) were incubated overnight at 4 °C with 2 μg anti-STK32C or anti-HSP90 antibodies plus 15 μL protein A/G agarose beads. Beads were washed and proteins eluted for Western blot analysis.

### Immunofluorescence

HCT116 cells grown on coverslips were fixed, permeabilized, blocked, and incubated with anti-STK32C and anti-HSP90 antibodies overnight at 4 °C. Secondary antibodies conjugated with Alexa Fluor dyes were applied for 2 h at room temperature. Cells were counterstained with DAPI and imaged using FLUOVIEW FV10i confocal microscope.

### Computational docking and homology analysis

Crystal structures of HSP90 (PDB: 2CG9) and STK32C (UniProt: Q86UX6) were visualized and aligned using PyMOL. Molecular docking simulations were conducted to predict binding interfaces, affinity, and key interactions including hydrogen bonding and hydrophobic contacts.

### HSP90 fragment expression and purification

GST-tagged HSP90 fragments (N, M, C domains) were expressed in DH5α E. coli, induced with IPTG, lysed, and purified by sonication and centrifugation as described.

### Coomassie blue staining

Eluted proteins were separated on 15% SDS-PAGE gels, stained with 0.1% Coomassie Blue R-350 for 2 h, destained, and imaged.

### GST pull-down assay

Purified GST-HSP90 fragments were incubated with HCT116 lysates and bound to glutathione magnetic beads. After washing, bound proteins were eluted and analyzed by Western blotting.

### Lentiviral infection and stable cell line generation

STK32C shRNA or control shRNA cloned in pGIPZ/GFP + Puro vector was packaged into lentivirus using HEK293T cells. HCT116 cells were infected and selected with puromycin (100 μg/mL) for 2 weeks. Knockdown efficiency was confirmed by Western blot.

### Xenograft tumor model

Animal experiments were approved by Kyung Hee University IACUC (protocol KHUASP(SE)23-292). BALB/c nude mice (5 weeks old) were subcutaneously injected with 1×10⁶ HCT116 cells expressing control or STK32C shRNA. Tumor volumes were measured with calipers and calculated as (length × width × height)/2. After 30 days, mice were sacrificed, tumors excised for weight measurement, Western blot, and IHC analysis (STK32C, HSP90, AKT1, PCNA, Cleaved-Caspase-3).

### Statistical analysis

Data are presented as mean ± standard deviation (SD). Statistical significance was evaluated by Student's t-test or one-way ANOVA using SPSS 19.0 and GraphPad Prism 8.0. Survival analyses were performed with TCGA datasets and Kaplan-Meier method. P values <0.05 were considered significant.

## Results

### Poor prognosis of STK32C overexpression and its endogenous levels in normal cells and colorectal cancer cells

**Figure 1:**
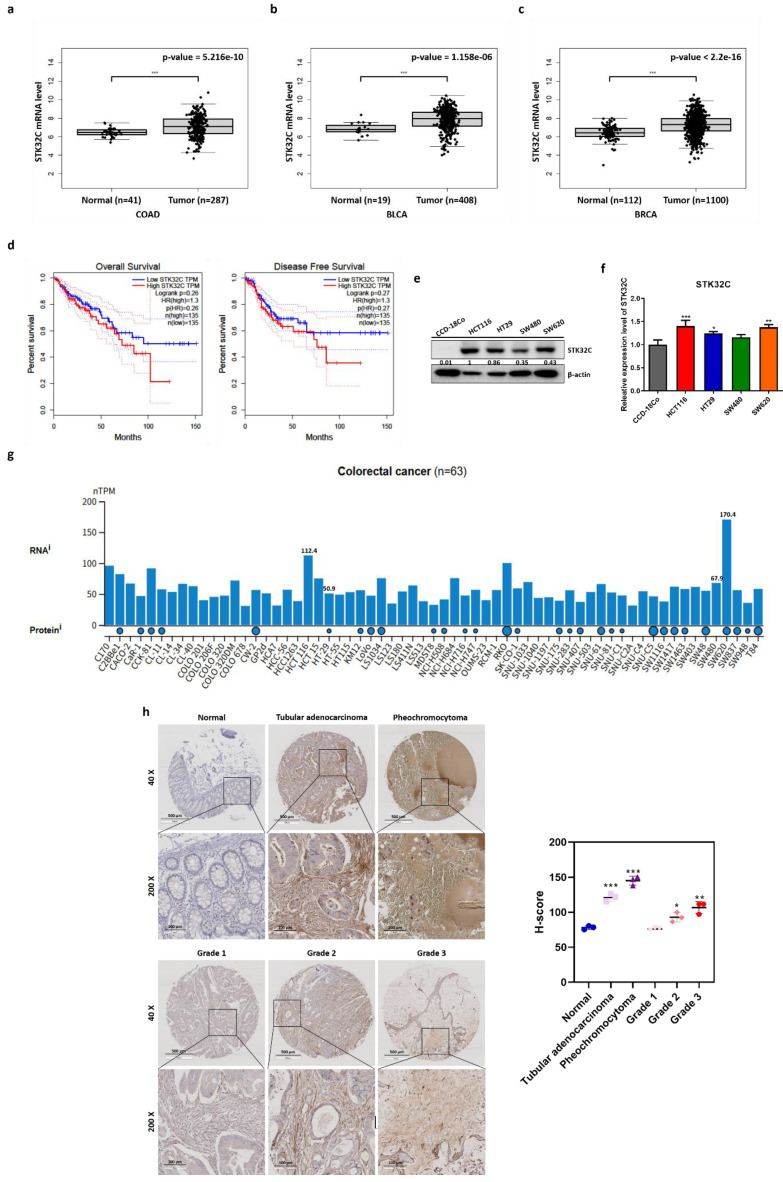
** Poor prognosis of STK32C overexpression and its endogenous levels in normal tissues and CRCs. (a)** mRNA expression level of STK32C in CRCs (n=287) and normal tissues (n= 41). **(b)** mRNA expression level of STK32C in bladder cancer (BLCA; tumor n = 408) and normal n = 19). mRNA expression level of STK32C in breast cancer (BRCA; tumor n = 1100) and normal (n = 112)). **(d)** Effect of STK32C expression on OS and DFS in colorectal cancer patients with low (n=135) and high (n=135) STK32C expression levels based on TCGA data. **(e)** Endogenous expression levels of STK32C in CCD-18co, HCT116, HT29, SW480 and SW620 cells. **(f)** Bar graph on endogenous expression levels of STK32C in CCD-18co, HCT116, HT29, SW480 and SW620 cells. * p<0.05, **<0.01, ***<0.001 vs normal cells. n=3. **(g)** STK32C mRNA expression across colorectal cancer cell lines (n = 63) from the Human Protein Atlas (HPA) dataset, presented as normalized transcripts per million (nTPM). **(h)** IHC staining of STK32C in human CRC patient tissues and normal tissues. Scale bar, 100 μm. n=3.

Bioinformatics analysis was conducted with TCGA dataset to identify STK32C expression level in colon, bladder and breast cancers and normal tissues. Here STK32C was significantly (p<0.001) overexpressed at mRNA level in CRC tissues (BRCA; tumor n = 287, normal n = 41). (Figure 1a), bladder cancer tissues (BLCA; tumor n = 404, normal n = 28) (Figure 1b) and breast cancer tissues (BRCA; tumor n = 1085, normal n = 291). (Figure 1c) compared to normal tissues. Although the expression levels varied across individual samples, both cohorts displayed a trend of higher STK32C expression in tumor tissues compared with normal controls. Also, DFS and OS rates were longer in CRC patients with low expression of STK32C compared to those with its high expression (Figure 1d) by Kaplan Meier analysis. Of note, high expression of STK32C was observed in HCT116 and SW620, while relatively low expression was detected in HT29 and SW480 cells (Figure 1e-f). Publicly available datasets showed variable endogenous expression of STK32C among colorectal cancer cell lines (n = 63). RNA-seq data (nTPM) revealed relatively high expression in SW620 and HCT116, whereas moderate to low levels were detected in HT29 and SW480. Protein abundance data were consistent with these findings, supporting the heterogeneity of STK32C expression across CRC models (Figure 1g). Consistently, tissue array reveals highly expression of STK32C in tubular adenocarcinoma and pheochromocytoma of CRCs compared to normal tissues. Additionally, tumor stages were in parallel with upregulation of STK32C (Figure 1h).

### STK32C depletion exerts cytotoxic, antiproliferative and antiinvasive activity in CRCs with its decreased stability

**Figure 2:**
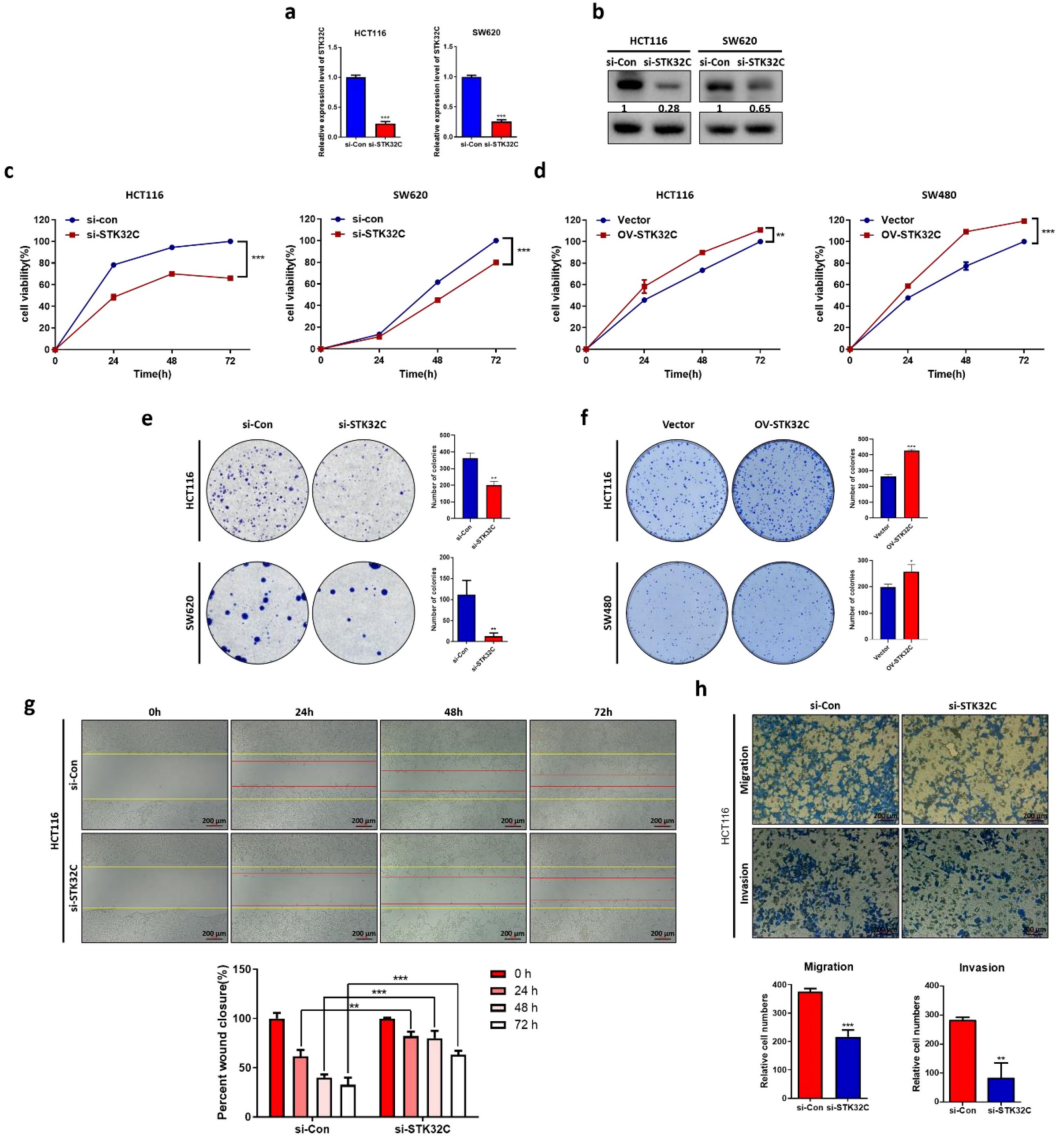
**Effect of STK32C depletion on cytotoxicity, proliferation, migration, invasion and its stability in colon cancer cells. (a)** STK32C mRNA expression in HCT116 and SW620 cells (***<0.001). n=3. **(b)** STK32C protein expression in HCT116 and SW620 cells. n=3. **(c)** Effect of STK32C depletion on the cytotoxicity in HCT116 and SW620 cells (*** < 0.001 vs untreated control). n=3. **(d)** Effect of STK32C depletion on the colonies of HCT116 and SW620 cells (**<0.01, ***<0.001 vs untreated control). n=3. **(e)** Effect of STK32C depletion on migratory activity in HCT116 cells by wound healing assay. Scale bar, 200 μm (**<0.01, ***<0.001 vs untreated control). n=3. **(f)** Effect of STK32C depletion on invasive activity in HCT116 cells by invasion assay. Scale bar, 200 μm (**<0.01, ***<0.001 vs untreated control). n=3. Experiments were performed in triplicate and repeated three times.

STK32C was successfully depleted at the mRNA and protein levels in HCT116 and SW620 cells (Figure 2a-b). Also, STK32C depletion significantly reduced the viability of HCT116 and SW620 cells compared to untreated control by MTT assay (Figure 2c). Also, STK32C depletion significantly inhibited the number of colonies in HCT116 and SW620 cells by colony formation assay (Figure 2d). In addition, wound healing assay reveals that STK32C depletion hindered the migratory capacity of HCT116 cells (Figure 2e). Additionally, STK32C depletion significantly inhibited the invasive activity of HCT116 cells by transwell plate assay (Figure 2f).

### Differentially expressed gene profile and signaling pathways in STK32C depleted HCT116 cells

**Figure 3:**
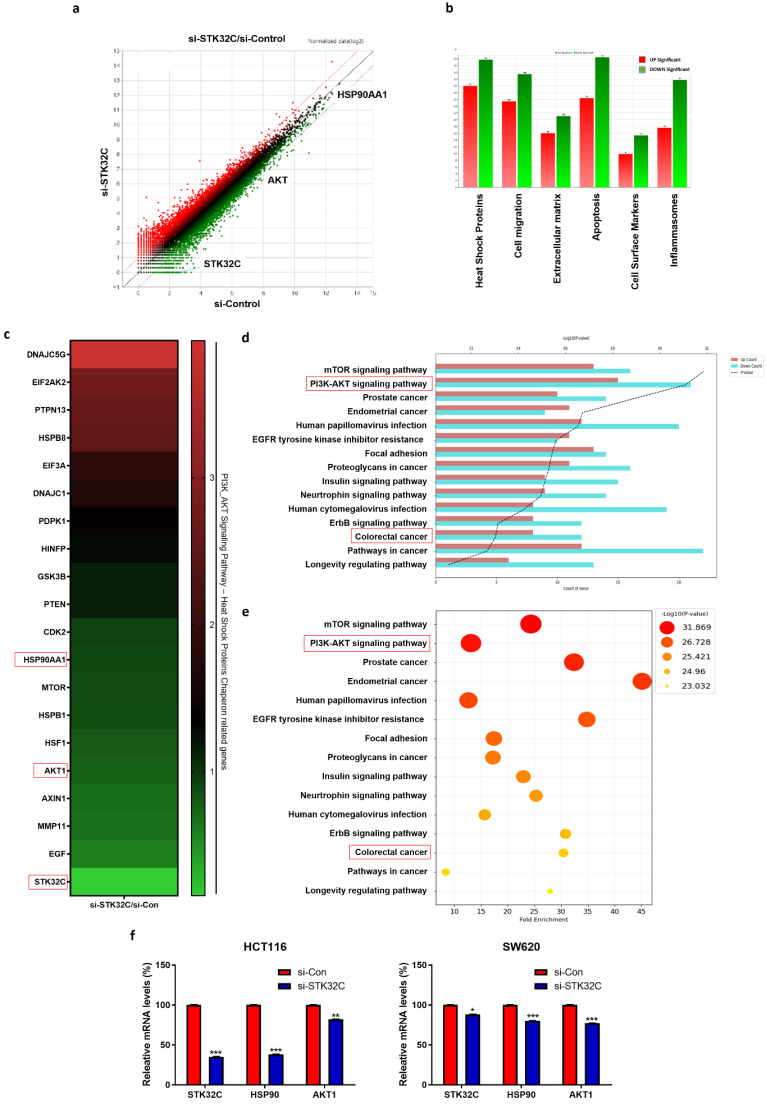
**Effect of STK32C depletion on the gene profile and signaling pathways in HCT116 cells and its validation for HSP90 and AKT1 in HCT116 and SW620 cells by qRT-PCR. (a)** Scatter plot in STK32C depleted HCT116 cells. **(b)** Effect of STK32C depletion on signaling pathways in STK32C depleted HCT116 cells. **(c)** Heatmap in HCT116 and SW620 cells (Red; upregulation; Green: downregulation). (d-e) Effect of STK32C depletion on signaling pathways (percentage) in STK32C depleted HCT116 cells. **(f)** Effect of STK32C depletion on STK32C, HSP90, and AKT1 in HCT116 and SW620 cells (*<0.05, **<0.01, ***<0.001 vs untreated control). n=3. Experiments were performed in triplicate and repeated.

NGS analysis reveals that there are differentially expressed gene profiles at the heat map (Red; upregulation; Green: downregulation) (Figure 3a,3c). Herein DNAJC5G, EIF2AK2, PTPN13, HSPB8, EIF3A, DNAJC1, PDPK1, HINFP, GSK3B and PTEN genes were upregulated, while CDK2, HSP90AA1, MTOR, HSPB1, HSF1, AKT1, AXIN1, MMP11, EGF and STK32C were downregulated in STK32C depleted HCT116 cells (Table 1), implying the association of HSP90 and PAM signaling.

GO analysis shows that STK32C depletion affects the relevant signaling such as Heat Shock Protein (67.78%), Apoptosis (64.84%), Cell migration (58.93%), Inflammasomes (49.41%), Extracellular matrix (37.12%), and Cell Surface Markers (25.27%) (Figure 3b), which indicates the percentages for differentially expressed genes mapped to each pathway relative to the total number of identified DEGs. Also, PI3K/AKT/mTOR signaling and HSP90 are mainly involved in STK32C depleted HCT116 cells (Figure 3d, 3e). Thus, we validated the effect of STK32C depletion on HSP90 and AKT1 in HCT116 and SW620 cells. Herein RT-qPCR shows that STK32C depletion downregulates the expression of HSP90 and AKT1 in HCT116 and SW620 cells (Figure 3f).

### Effect of STK32C depletion on apoptosis in HCT116 and SW620 cells

**Figure 4:**
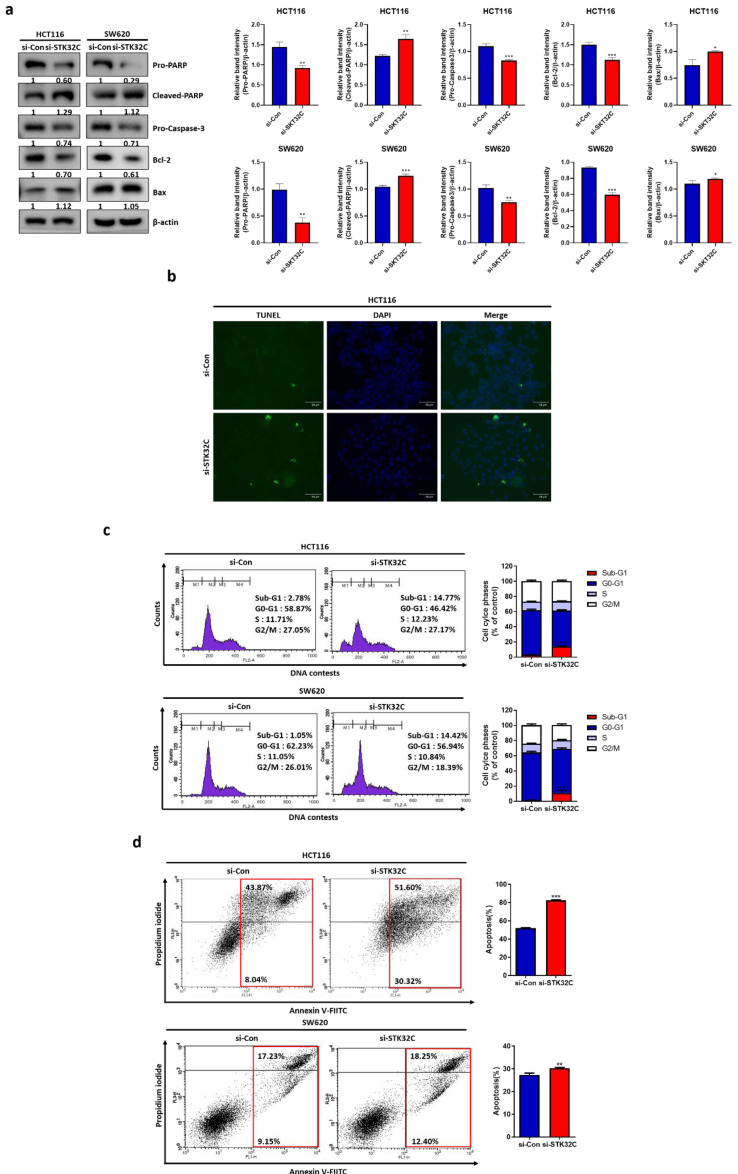
** Effect of STK32C depletion on apoptosis in HCT116 and SW620 cells. (a)** Effect of STK32C depletion on apoptosis related proteins in HCT116 and SW620 cells. (* <0.05, **<0.01, ***<0.001 vs untreated control). n=3. **(b)** Effect of STK32C depletion on TUNEL positive bodies in HCT116 cells. Scale bar, 50 μm. n=3. **(c)** Effect of STK32C depletion on sub-G1 population in HCT116 cells. n=3. **(d)** Effect of STK32C depletion on apoptotic portion in HCT116 cells by staining with Annexin V and propidium iodide (**<0.01, ***<0.001 vs untreated control). n=3. Experiments were performed in triplicate and repeated three times.

Silencing of STK32C attenuated the expression of pro-PARP, pro-Caspase-3, and Bcl-2, increased cleaved-PARP and Bax (Figure 4a). Also, STK32C depletion increased the number of green fluorescent TUNEL positive bodies compared to untreated control (Figure 4b). Consistently, STK32C depletion increased sub-G1 population compared to untreated control in HCT116 and SW620 cells (Figure 4c). Likewise, STK32C depletion increased apoptotic portion stained by Annexin V and propidium iodide compared to untreated control in HCT116 (Figure 4d) and SW620 cells (Figure 4e).

### Effect of STK32C depletion or overexpression on HSP90 and their binding and colocalization in CRCs

**Figure 5:**
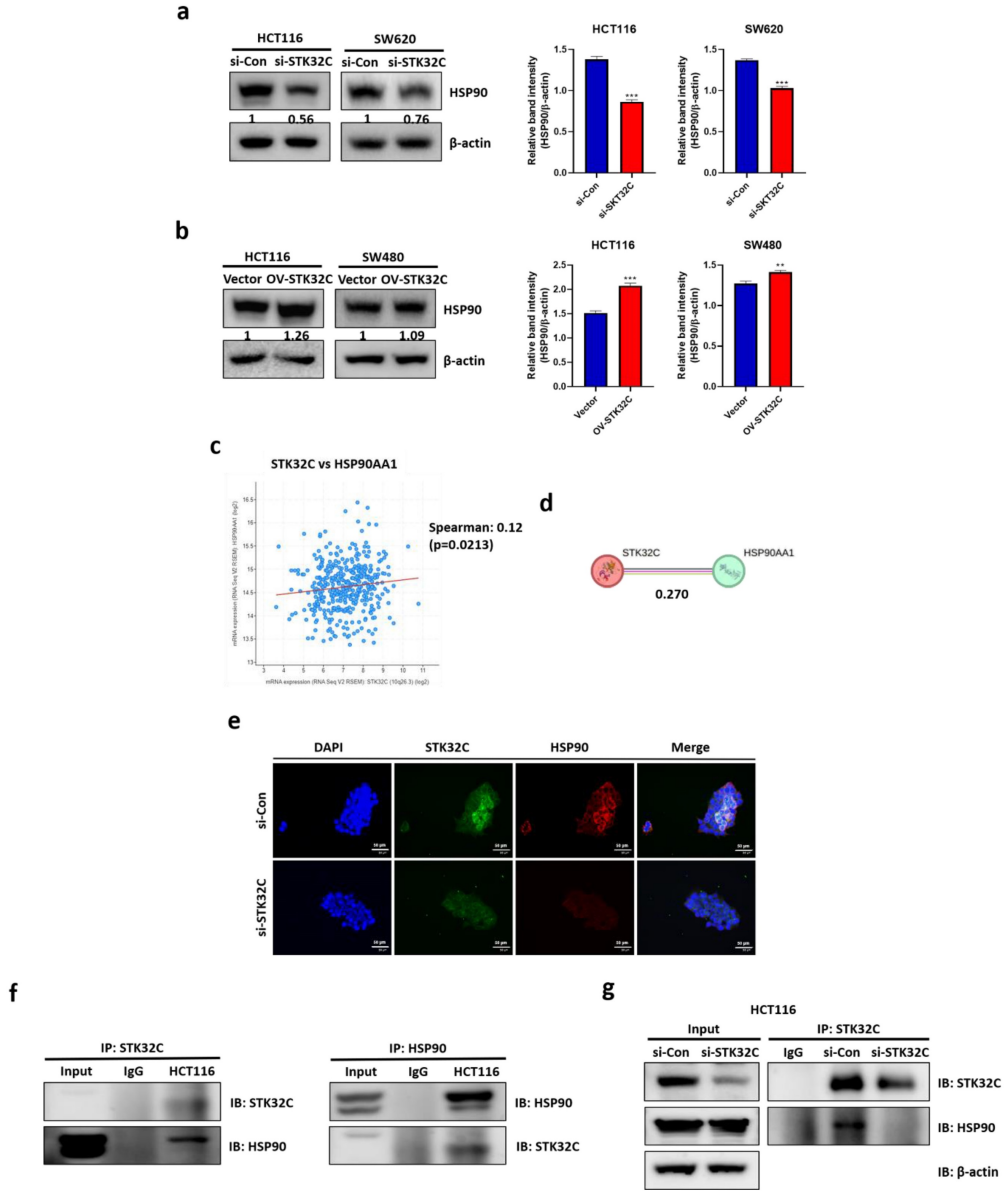
**Effect of STK32C depletion or overexpression on HSP90 and their binding and colocalization in HCT116 cells. (a)** Effect of STK32C depletion on HSP90 in HCT116 and SW480 cells. (***<0.001 vs untreated control). **(b)** Effect of STK32C overexpression on HSP90 in HCT116 and SW480 cells. ( **<0.01, ***<0.001 vs untreated control). **(c)** Spearman correlation efficient (r=0.12) between STK32C and HSP90 at mRNA level by cBioPortal database analysis. **(d)** PPI (r=0.270) between STK32C and HSP90 by String database. **(e)**Colocalization between STK32C and HSP90 in HCT116 cells by Immunofluorescence. Scale bar, 50 μm. n=3. **(f)** Binding between STK32C and HSP90 in HCT116 cells by Immunoprecipitation. n=3. **(g)** Effect of STK32C depletion on the binding to HSP90 in HCT116 cells by Immunoprecipitation. n=3. Experiments were performed in triplicate and repeated three times.

Consistently, STK32C depletion downregulated HSP90 in HCT116 and SW620 cells (Figure 5a), while STK32C overexpression upregulated HSP90 in HCT116 and SW480 cells by Western blotting (Figure 5b). Regarding the interaction between STK32C and HSP90, cBioPortal database reveals a positive correlation between STK32C and HSP90 at mRNA level in colorectal cancer (P < 0.01, r = 0.12) (Figure 5c). The predicted protein-protein interaction (PPI) score between STK32C and HSP90AA1 was 0.270, as obtained from the STRING database (Figure 5d). Consistently, Immunofluorescence shows that STK32C is colocalized into HSP90 in HCT116 cells (Figure 5e). Furthermore, Immunoprecipitation demonstrates a direct interaction between STK32C and HSP90 (Figure 5f). Also, STK32C depletion disrupts the binding of STK32C and HSP90 in HCT116 cells (Figure 5g).

### Binding site and homology between STK32C and HSP90 domain and effect of STK32C depletion on HSP90 chaperone proteins in HCT116 cells

**Figure 6:**
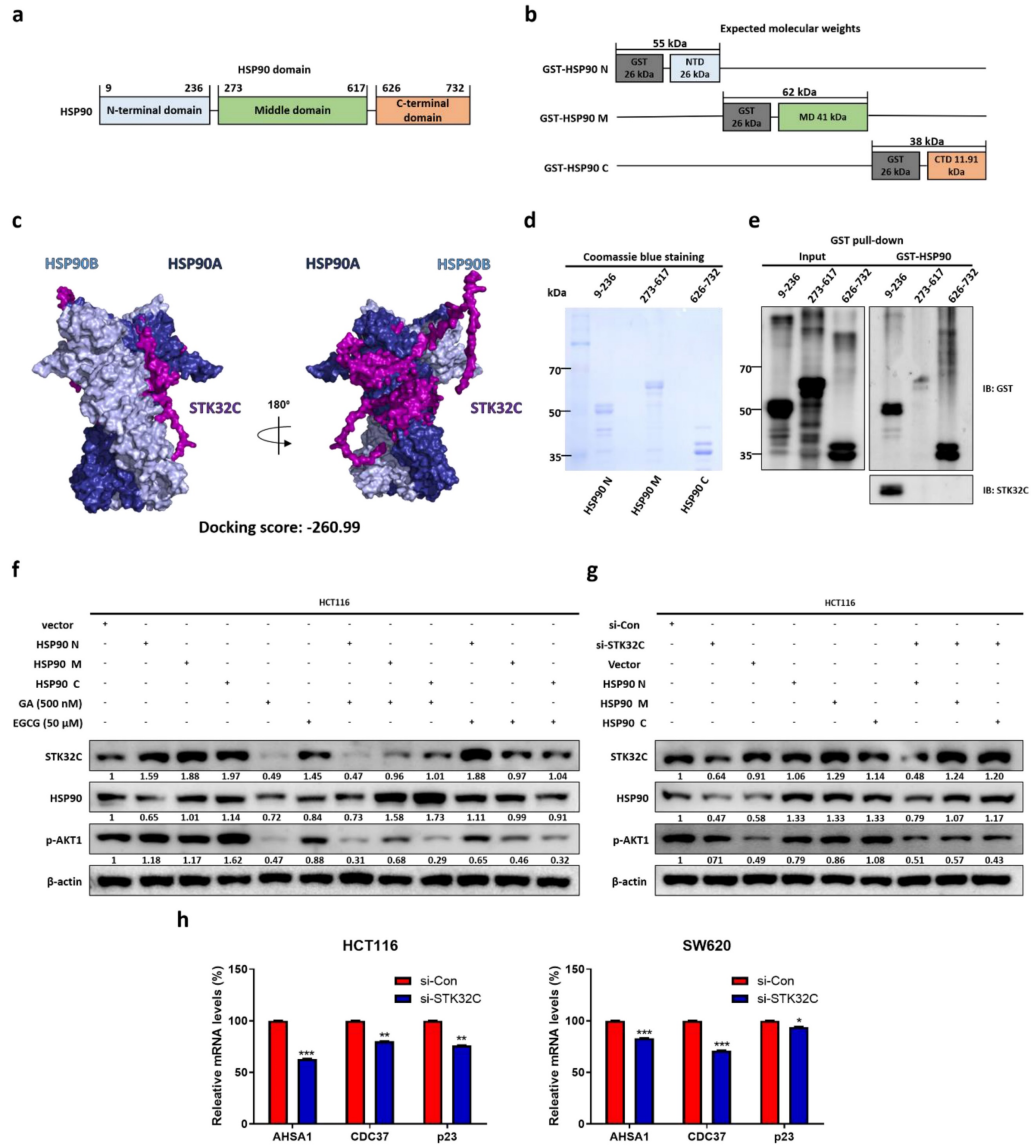
** Binding site and homology between STK32C and HSP90 domain and effect of STK32C depletion on HSP90 chaperone proteins in HCT116 cells. (a)** HSP90 three domain sizes. **(b)** Molecular weights of GST tagged HSP90 domains. **(c)** Homology analysis of STK32C 3D structure (PDB Q86UX6) and HSP90 3D structure (PDB 2CG9) by using pyMOL software. Deep blue; HSP90α, light blue: HSP90β, purple: STK32C. **(d)** Sizes of three HSP90 domain fragments by Coomassie blue staining. n=3. **(e)** Binding site of STK32C to HSP90 domain by GST pull-down assay. n=3. **(f)** Effect of HSP90 N-terminal inhibitor GA (500 nM) or HSP90 C-terminal inhibitor EGCG (50 μM) on STK32C and HSP90 domain fragments in HCT116 cells. n=3. **(g)** Effect of STK32C depletion on HSP90 domain fragments in HCT116 cells. n=3. **(h)** Effect of STK32C depletion on AHSA1, CDC37 and p23 as co-chaperone proteins in HCT116 and SW620 cells by RT-qPCR (*<0.05, **<0.01, ***<0.001 vs untreated control). n=3. Experiments were performed in triplicate and repeated three times.

HSP90 domains include three domain regions: domain sizes of the N-terminal domain t(9-236), the middle domain (273-617), and the C-terminal domain (626-732) (Figure 6a) and their different molecular weights such as HSP90N (55 kDa), HSP90M (62 kDa), and HSP90C (38 kDa) shown in HSP90 domain mapping (Figure 6b). Homology modeling indicates that STK32C 3D structure (PDB Q86UX6) matches to HSP90α 3D structure (PDB 2CG9) rather than HSP90β structure by using pyMOL software (Figure 6c). Consistently, computational docking analysis^27^ for HSP90 (PDB ID: 2CG9) and STK32C (UniProt ID: Q86UX6) revealed a stable interaction mediated by hydrogen bonding and hydrophobic forces. Structural homology analysis using PyMOL confirmed their similarity and identified potential binding interfaces. Key molecular interactions at the binding interface included hydrogen bonds involving VAL118, GLY121, ARG380, and MET84, with bond distances ranging from 2.27 to 3.38 Å. Among these, GLY121 (2.27 Å) formed the strongest interaction, followed by ARG380 (2.57 Å) and VAL118 (2.91 Å), indicating a high binding affinity. Additionally, hydrophobic interactions, including Pi-Sigma and Pi-Alkyl bonds at MET84 and ALA41 (3.79-5.49 Å), contributed to the overall stability of the complex. Also, *Protein-protein docking.* monomer structures of HSP90 (PDB 2CG9, chain A; cofactors removed) and STK32C (AlphaFold model AF-Q86UX6-F1**)** were docked by HDOCK SERVER 2.4. The top-ranked docking pose showed a docking score of -260.99 (kcal/mol) with a confidence score of 0.902. Also, top-ranked clusters were selected by native scoring and cluster size. Binding free energy (ΔG) was -4.3 (kcal/mol^-1^), while Kd(M) was 8.6 × 10⁻⁴ at 37 ℃, which was analyzed in interface contacts and buried surface area by PRODIGY (Table 2).

Visualization through interaction mapping tools confirmed that hydrogen bonding serves as the primary stabilizing force, while hydrophobic interactions enhance the structural integrity of STK32C within the HSP90 binding pocket, suggesting a strong and specific binding interaction. After the sizes of three HSP90 domain fragments were confirmed by Coomassie blue staining (Figure 6d), GST pull-down assay was conducted in HCT116 cells. Notably, STK32C binds to N-terminal of HSP90 rather than other domains of HSP90 in HCT116 cells by co-immunoprecipitation (Figure 6e). Furthermore, the effect of HSP90 N-terminal inhibitor Ganetespib (GA)^28^, ^29^ or HSP90 C-terminal inhibitor epigallocatechin gallate (EGCG)^30^ was evaluated on STK32C and HSP90 domain fragments in HCT116 cells. As expected, GA reduced the expression of N terminal of HSP90, AKT1 and STK32C more than M or C-terminal of HSP90, while EGCG attenuated the expression of C-terminal of HSP90 more than N or M-terminal of HSP90, without affecting STK32C and p-AKT1 (Figure 6f). Conversely, STK32C depletion reduced the expression of N terminal of HSP90, AKT1 than more than N or M-terminal of HSP90 in HCT116 cells (Figure 6g). Consistently, STK32C depletion significantly downregulated the expression of co-chaperone proteins^31^, ^32^ including activator of HSP90 ATPase activity 1(AHSA1), cell division cycle 37 (CDC37), and p23 in HCT116 and SW620 cells (Figure 6h).

### STK32C regulates PAM signaling as an upstream of AKT in HCT116 cells

**Figure 7:**
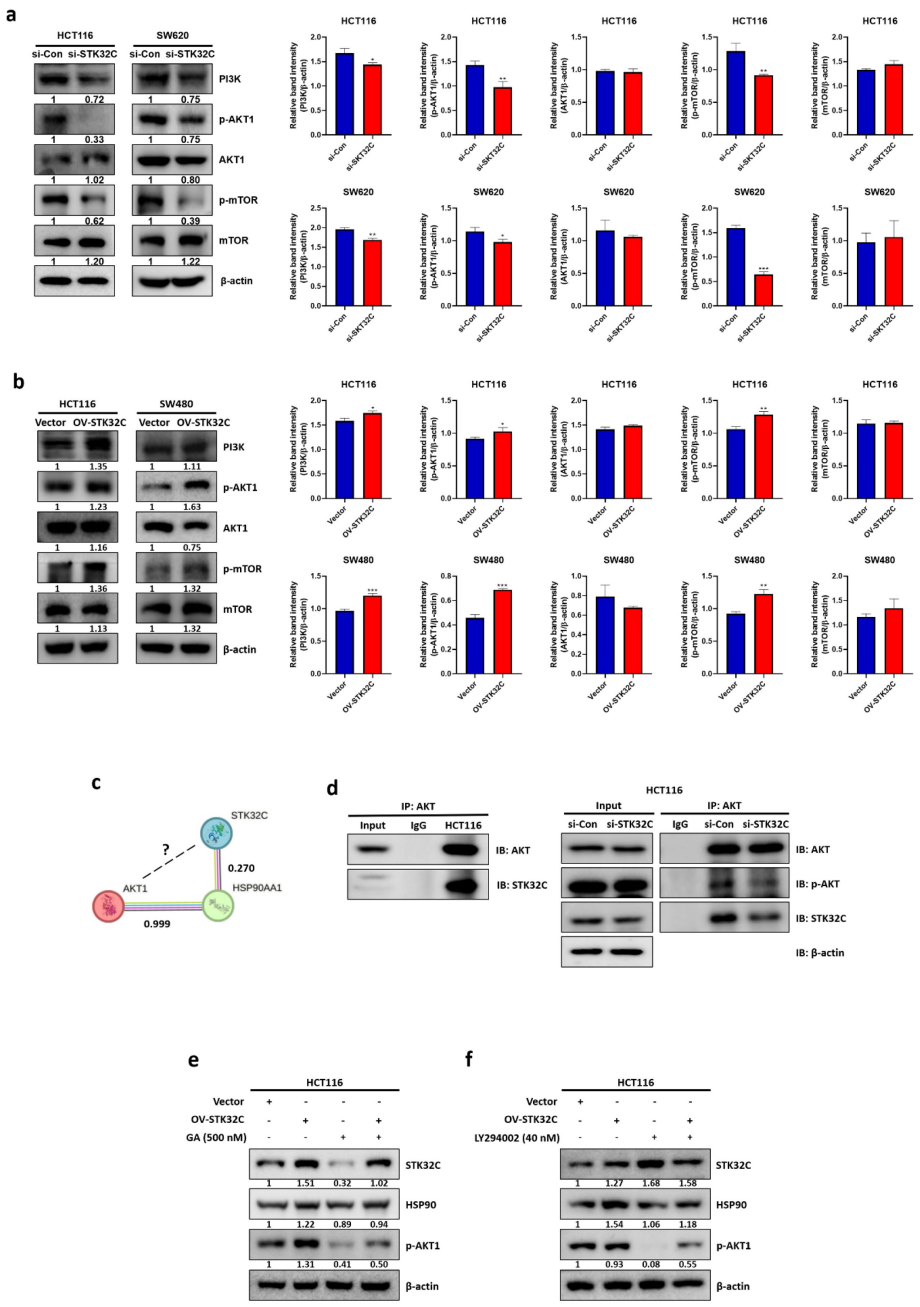
** Effect of STK32C on PAM signaling pathway in CRCs. (a)** Effect of STK32C depletion on PAM signaling in HCT116 and SW620 cells. (*<0.05, **<0.01, ***<0.001 vs untreated control). n=3. **(b)** Effect of STK32C overexpression on PAM signaling in HCT116 and STK32C lowly expressed SW480 cells. (*<0.05, **<0.01, ***<0.001 vs untreated control). n=3. **(c)** PPI score between STK32C and HSP90 or AKT1. **(d)** Co-immunoprecipitation analysis showing the physical interaction between AKT1 and STK32C in HCT116 cells. **(e)** Effect of HSP90 inhibitor GA and/or STK32C overexpression on HSP90 and p-AKT in HCT116 cells. n=3. **(f)** Effect of AKT inhibitor LY294002 and/or STK32C overexpression on HSP90 and p-AKT in HCT116 cells. n=3. Experiments were performed in triplicate and repeated three times.

In the current work to assess the effect of STK32C on PAM signaling pathway including PI3K, p-AKT1, and p-mTOR, STK32C depletion attenuated the expression of PI3K, p-AKT, p-mTOR, and HSP90 in HCT116 and SW480 cells (Figure 7a). In contrast, STK32C overexpression enhanced the expression of PI3K, p-AKT, p-mTOR, and HSP90 in HCT116 and SW480 cells (Figure 7b). However, the binding score between STK32C and AKT1 was not elucidated to date, though HSP90 directly binds to AKT1 with PPI interaction score of 0.99 by STRING database (Figure 7c), which was supported by IP that STK32C binds to AKT in HCT116 cells (Figure 7d). Interestingly, HSP90 inhibitor GA or STK32C depletion attenuated the expression of HSP90, p-AKT in HCT116 cells, while AKT inhibitor LY294002 did not affect STK32C, despite its downregulation of HSP90 and p-AKT (Figure 7e,7f), implying that STK32C works as an upstream of AKT1.

### Synergistic effect of STK32C depletion with 5-FU in HCT116 cells

**Figure 8:**
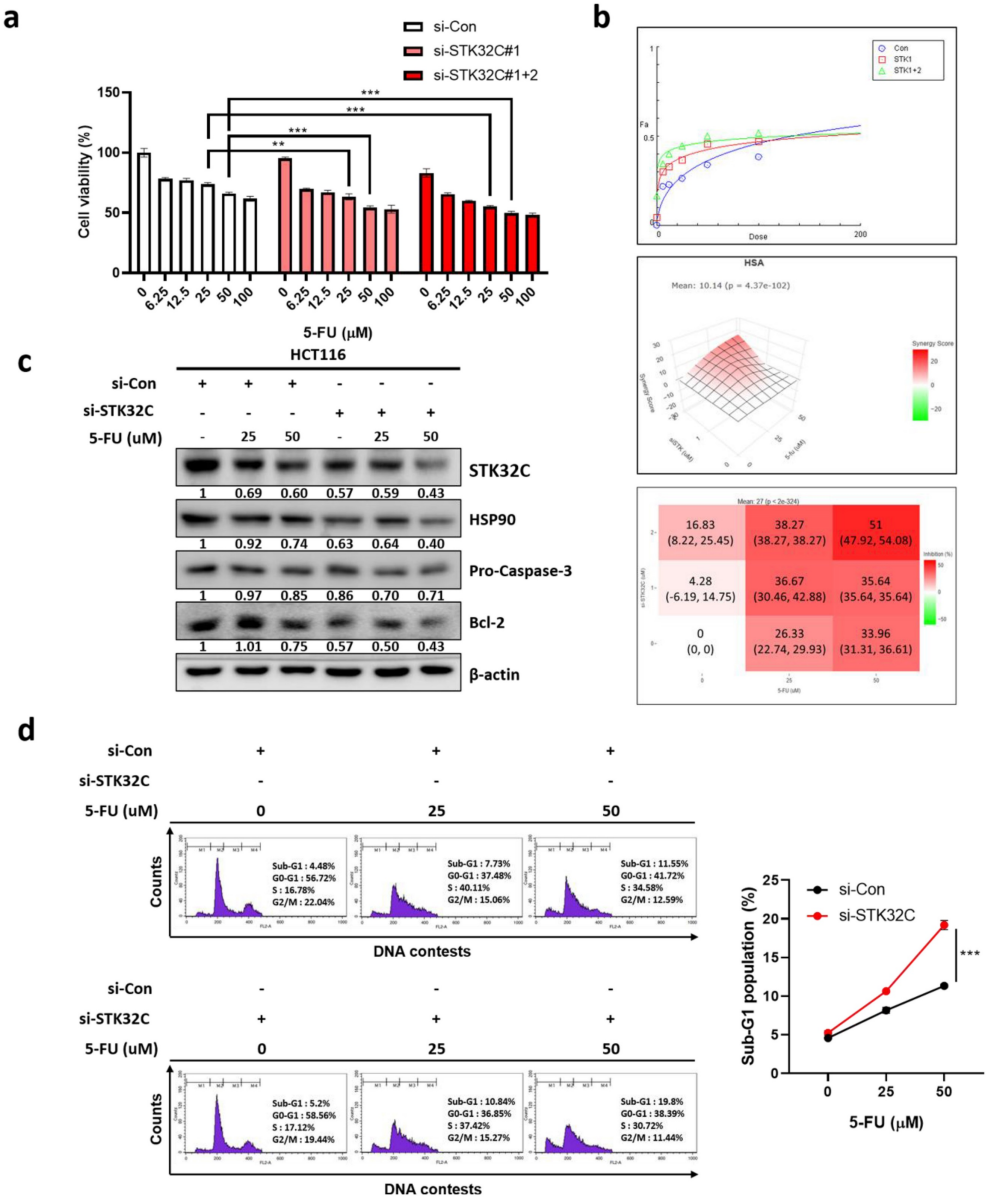
** Synergistic effect of STK32C depletion and 5-FU in HCT116 cells (a)** Effect of STK32C depletion and 5-FU combination on the cytotoxicity in HCT116 cells by MTT assay (**<0.01, ***<0.001 vs 5-FU alone control). n=3. **(b)** Synergy analysis between STK32C depletion and 5-FU by using CompuSyn and SynergyFinder software. Synergy is determined when the combination index (CI) is below 1, and the synergy score exceeds 10 in specific concentration ranges. **(c)** Effect of STK32C depletion and 5-FU combination on STK32C, HSP90, caspase-3, and Bcl-2 in HCT116 cells by Western blotting. n=3. **(d)** Effect of STK32C depletion and 5-FU combination on the Sub-G1 population. ***<0.001 vs untreated control. n=3. Experiments were performed in triplicate and repeated three times.

To assess the synergistic potential of STK32C depletion with 5-FU, MTT assay, Western blotting, and cell cycle analysis were conducted in HCT116 cells. STK32C depletion significantly enhanced the cytotoxic effect of 5-FU in HCT116 cells (Figure 8a). It was further confirmed by CompuSyn analysis that the combination index (CI) was below 1 for synergy, and by SynergyFinder analysis that a synergy score was over 10 for synergy in specific concentration ranges with the red-colored region (Figure 8b). Consistently, STK32C depletion potentiated the inhibitory effect of 5-FU on HSP90, procaspase 3 and Bcl-2, implying the synergistic apoptosis by combination of STK32C depletion and 5-FU (Figure 8c). Furthermore, STK32C depletion significantly increased Sub-G1 population to 10.84% and 19.8% with 5-FU at 25 μM and 50 μM, respectively, compared to 7.73% and 11.55% in untreated control (Figure 8d). These findings indicate that STK32C depletion enhances the apoptotic activity of 5-FU in HCT116 cells.

### STK32C depletion suppressed the growth of HCT116 cells inoculated in BALB/c nude mice, with reduced expression of STK32C, HSP90, AKT1, and PCNA, and increased expression of cleaved-caspase3

**Figure 9:**
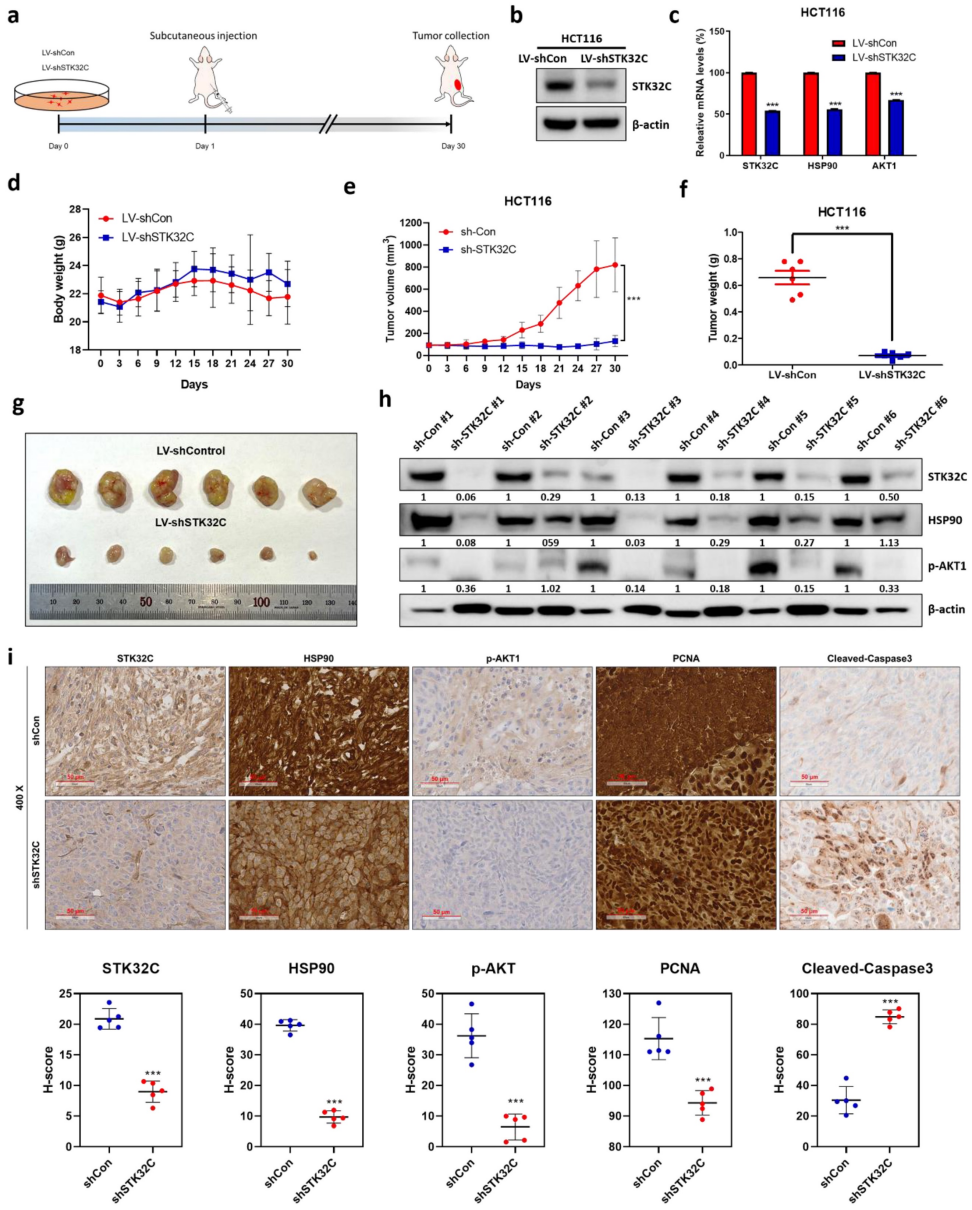
** Effect of STK32C depletion on the growth of HCT116 cells and IHC expression of STK32C, HSP90, AKT1 and PCNA in BALB/c nude mice. (a)** Scheme of animal study plan. **(b)** Protein expression of HCT116 cells transfected with Lv-shCon or Lv-shSTK32C plasmids. **(c)** mRNA expression of HCT116 cells transfected with Lv-shCon or Lv-shSTK32C plasmids (***<0.001vs untreated control). **(d)** Body weights in control (n=6) and STK32C depletion (n=6) groups. **(e)** Tumor volumes in control (n=6) and STK32C depletion (n=6) groups. (***<0.001 vs untreated control). **(f)** Tumor weights in control (n=6) and STK32C depletion (n=6) groups. **(g)** Isolated tumors in control (n=6) and STK32C depletion (n=6) groups. **(h)** Protein expression of STK32C, HSP90, and p-AKT1 in control (n=6) and STK32C depletion (n=6) groups. **(i)** Effect of STK32C depletion on the expression of STK32C, HSP90, p-AKT1, and PCNA by Immunohistochemistry.

Animal study was performed for 30 days in HCT116 tumor xenograft model based on the animal study scheme (Figure 9a). STK32C expression was confirmed in HCT116 cells transfected with Lv-shCon or Lv-shSTK32C plasmid by Western blotting (Figure 9b) and qRT-PCR (Figure 9c). The body weight of the experimental animals was not affected in all groups, indicating no toxicity by STK32C depletion (Figure 9d). Consistently, tumor volume (Figure 9e) and weights (Figure 9f) were significantly suppressed in STK32C depletion group compared to sham control group. Also, STK32C depletion reduced the growth of HCT116 cells in BALB/c nude mice compared to untreated control (Figure 9g), which was confirmed in tumor tissues by Western blotting (Figure 9h). Furthermore, immunohistochemistry revealed that STK32C depletion attenuated the expression of STK32C, HSP90, AKT1, and PCNA which are markers of proliferation and survival, and increased the expression of cleaved-Caspase3 (Figure 9i).

## Discussion

In the present study, the underlying oncogenic mechanism of STK32C was explored in colorectal cancers, based on previous evidence that STK32C is highly expressed in bladder cancer^24^ and also is a molecular target for doxorubicin resistance in breast cancer^33^. TCGA analysis reveals that STK32C was overexpressed with poor prognosis in Overall Survival and Disease-Free Survival rates, which was supported by our data that STK32C was endogenously overexpressed in HCT116, HT29, SW480, and SW620 CRCs compared to CCD-18Co normal fibroblast cells, implying oncogenic potency of STK32C. Conversely, negative regulation of STK32C showed cytotoxicity, antimigratory and antiinvasive effects in HCT116 and SW620 cells, indicating antimetastatic activity of STK32C depletion in CRCs. Also, STK32C depletion inhibited the expression of pro-PARP, pro-Caspase-3, and Bcl-2 and increased Bax, sub-G1 population, Annexin V apoptotic portion and TUNEL positive cells in HCT116 and SW620 cells, demonstrating apoptotic effect of STK32C depletion. Indeed, NGS analysis showed that PI3K/AKT/mTOR and HSP90 were downregulated in STK32C depleted HCT116 cells, which was validated by RT-qPCR analysis.

Among heat-shock protein families of HSP100, 90, 70, 60, and the small HSP^33^, HSP90 as a vital chaperone protein is critically involved in tumor progression with three main conserved domains as the N-terminal domain, the middle domain, and the C-terminal domain^15^. Interestingly, computational structure prediction method and homology analysis reveal that STK32C 3D structure (PDB Q86UX6) matches to HSP90α 3D structure (PDB 2CG9) rather than HSP90β structure via stable interaction mediated by hydrogen bonding and hydrophobic forces, implying the close binding affinity between them. Furthermore, GST-pulldown assay shows that STK32C directly binds to HSP90 N-terminal domain (626-732) rather than middle domain or C-terminal of HSP90 after HSP90 fragment domains were confirmed by Coomassie blue staining. Consistently, the colocalization was observed between STK32C and HSP90 in HCT116 cells by Immunofluorescence, clearly demonstrating interaction between STK32C and HSP90. Also, HSP90 N-terminal inhibitor GA, not HSP90 C-terminal inhibitor EGCG, attenuated the expression of STK32C and p-AKT, while AKT inhibitor LY294002 did not affect STK32C in HCT116 cells, implying that STK32C works as an upstream of AKT. HSP90 co-chaperones are known as HSC70/HSP90-organizing protein (HOP), Peptidyl-prolyl cis-trans isomerases (PPIases), cell division cycle 37 (CDC37), activator of HSP90 ATPase homologue 1 (AHA1) and p23 (Sba1 in yeast)^34^. As expected, STK32C depletion attenuated mRNA expression of HSP90 co-chaperone proteins as AHSA1, CDC37 and p23 in HCT116 cells, indicating that STK32C modulates HSP90 chaperone machinery. In addition, STK32C depletion suppressed tumor sizes and volumes of HCT116 cells in BALB/c nude mice with decreased expression of STK32C, proliferating cell nuclear antigen (PCNA) as a proliferation marker, HSP90, p-AKT1 with no toxicity by Immunohistochemistry, suggesting antitumor effect of STK32C depletion *in vivo* via inhibition of HSP90 and AKT signaling axis. Recently, to reduce chemoresistance and to enhance the antitumor effect of chemotherapy are on the spotlight for effective cancer therapy^35^, ^36^. Recently, regarding gene depletion efficacy by siRNA transfection, Bao *et al.* suggested targeting YTHDF1 enhanced anti-PD-1 efficacy in CRCs^37^ and Wen *et al.* reported that cell migration and invasion were attenuated in ATP-citrate lyase (ACLY)-deficient HCT116 and RKO cell lines^38^.

Likewise, 5-FU that has been commonly prescribed for patients with CRCs has frequently failed to remove cancers due to its resistance^39^-^41^. In current work, combination of STK32C depletion and 5-FU significantly enhanced the cytotoxic effect, increased Sub-G1 population and attenuated expression of HSP90 and Bcl-2 and activated caspase-3 in HCT116 cells, demonstrating combinatorial potential of STK32C depletion with 5-FU with the necessity of animal study in the near future. In addition, the possible off-target effects by STK32C depletion can be expected such as p53/MDM2 in CRCs, since we acknowledge the limitation of using only two CRC cell lines, HCT116 (p53 wild-type) and SW620 (p53 mutant), and recognize the necessity of validating our findings across a broader panel of CRC models including patient-derived samples or clinical datasets. Also, future *in vivo* studies, including well-designed animal models and eventual human clinical trials, are essential to validate the therapeutic potential of STK32C targeting and assess its feasibility in clinical applications. Additionally, we propose the use of delivery platforms such as cRGD-modified lipid nanoparticles^42^ to enhance specificity and reduce *in vivo* degradation^10^, ^43^ of STK32C siRNA. Lastly, we highlight the potential for high-throughput screening to identify potent chemical or natural compounds that selectively inhibit STK32C, which may pave the way for the development of novel therapeutics suitable for clinical application.

**Figure 10:**
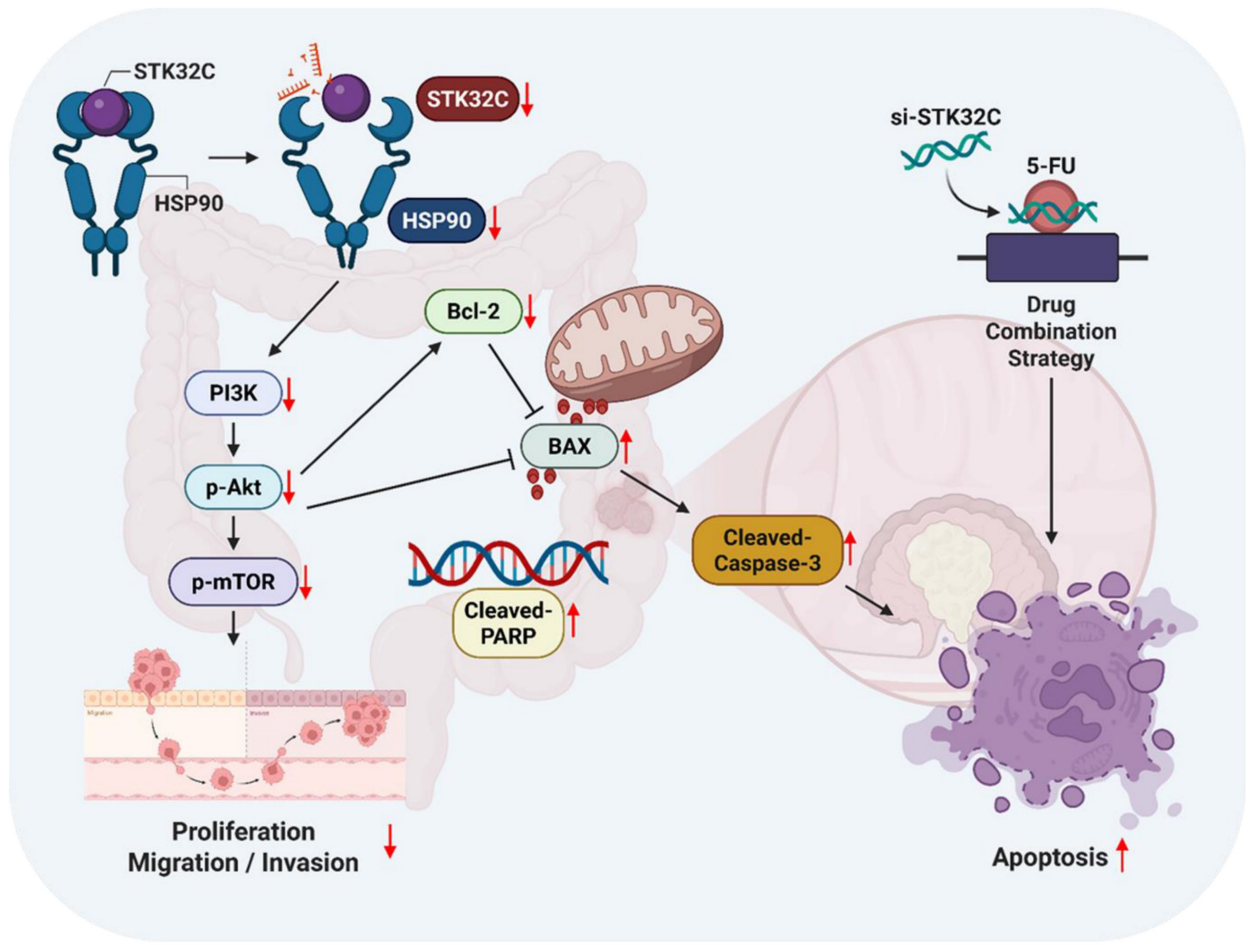
The schematic diagram on apoptotic effect of STK32C depletion in colon cancer and its combinatorial potential with 5-FU via HSP90 and PI3K/AKT/mTOR signaling axis.

In summary, in our current study, STK32C was overexpressed in HCT116, HT29, SW480, and SW620 colorectal cancer cells compared to CCD-18Co normal fibroblast cells, and its overexpression was associated with poor OS and DFS rates. STK32C depletion induced apoptosis and inhibited proliferation and invasion in HCT116 and SW620 cells. Notably, STK32C directly binds to the HSP90 N-terminal domain by molecular docking study, IP and GST-pulldown assay and is also colocalized with HSP90 in HCT116 cells. HSP90 N-terminal inhibitor GA, but not the HSP90 C-terminal inhibitor EGCG, reduced STK32C and p-AKT1 levels, while the AKT inhibitor LY294002 did not affect STK32C in HCT116 cells, implying that STK32C activates HSP90, which subsequently influences AKT as an upstream of AKT. Additionally, STK32C depletion reduced the growth of HCT116 cells in BALB/c nude mice with decreased expression of STK32C, HSP90, PCNA, and AKT, and increased expression of cleaved-Caspase3. Furthermore, combination of STK32C depletion and 5-FU significantly enhanced the cytotoxicity and Sub-G1 population and attenuated expression of HSP90 and Bcl-2 and activated caspase-3 in HCT116 cells. These findings support the novel evidence that targeting STK32C suppresses colon cancer progression and suggest its combinatorial potential with 5-FU via HSP90 and PI3K/AKT/mTOR signaling axis (Figure 10).

## Figures and Tables

**Table 1:** Differential gene expression profile in STK32C-depleted HCT116 cells.

Gene Symbol	Upregulation (folds)	Gene Symbol	Downregulation(folds)
DNAJC5G	3.932	CDK2	0.933
EIF2AK2	2.766	HSP90AA1	0.903
PTPN13	2.635	MTOR	0.873
HSPB8	2.501	HSPB1	0.869
EIF3A	1.861	HSF1	0.795
DNAJC1	1.695	AKT1	0.746
PDPK1	1.371	AXIN1	0.685
HINFP	1.253	MMP11	0.674
GSK3B	1.137	EGF	0.583
PTEN	1.127	STK32C	0.159

**Table 2:** Docking score, binding energy, and interaction profile between STK32C and HSP90.

Complex	Docking Score (kcal/mol)	Confidence Score	ΔG (kcal/mol^-1^)	Kd (M) at 37 ℃	Residue Name	Distance (Å)	Interaction Type	Bond Category
STK32C-HSP90	-260.99	0.902	-4.3	8.6 × 10⁻⁴	VAL118	2.91	Conventional Hydrogen Bond	Classical
					GLY123	2.64	Conventional Hydrogen Bond	Classical
					GLY121	2.27	Conventional Hydrogen Bond	Classical
					ARG380	2.57	Conventional Hydrogen Bond	Classical
					GLN119	3.38	Conventional Hydrogen Bond	Classical
					ASN37	2.6	Conventional Hydrogen Bond	Classical
					ASN37	2.78	Conventional Hydrogen Bond	Classical
					PHE124	2.99	Conventional Hydrogen Bond	Classical
					GLY100	2.57	Conventional Hydrogen Bond	Classical
					ASN92	2.76	Conventional Hydrogen Bond	Classical
					MET84	3.34	Conventional Hydrogen Bond	Classical
					THR171	3.36	Conventional Hydrogen Bond	Classical
					ASP79	3.29	Conventional Hydrogen Bond	Classical
					GLY121	2.75	Carbon Hydrogen Bond	Non-classical
					SER99	3.76	Carbon Hydrogen Bond	Non-classical
					ASN37	3.12	Carbon Hydrogen Bond	Non-classical
					ARG380	4.09	Active charge	Hydrophobic
					ARG380	4.92	Active charge	Hydrophobic
					MET84	3.79	Pi-Sigma	Hydrophobic
					MET84	5.49	Pi-Alkyle	Hydrophobic
					ALA41	4.17	Pi-Alkyle	Hydrophobic

## Abbreviations

Bax: BCL2 associated X
Bcl-2: B-cell lymphoma 2
PARP: Poly (ADP-ribose) polymerase
Caspase: Cysteine aspartyl-specific protease
CRC: Colorectal cancer
ECL: Enhanced chemiluminescence
EGFR: epidermal growth factor receptor
FACS: Fluorescence-activated cell sorting
FBS: Fetal Bovine Serum
mTOR: Mammalian target of rapamycin
MTT: 3-(4,5-dimethylthiazol-2-yl)-2,5-diphenyltetrazolium bromide
NGS: Next-generation sequencing
OD: Optical density
PAM: PI3K/AKT/mTOR
PBS: Phosphate Buffered Saline
PCNA: Proliferating cell nuclear antigen
PI: Propidium Iodide
PI3K: Phosphoinositide 3-kinase
SDS-PAGE: Sodium Dodecyl Sulfate-Polyacrylamide gel electrophoresis
STK32C: Serine/Threonine Kinase 32C
TUNEL: Terminal deoxynucleotidyl transferase dUTP nick end labeling
RT-qPCR: real-time quantitative polymerase chain reaction
GA: Ganetespib
EGCG: Epigallocatechin gallate
5-FU: 5-Fluorouracil
AHSA1: activator of HSP90 ATPase activity 1
CDC37: cell division cycle 37
